# Hybrid Approach for Multiple Visceral Aneurysms with Isolated Dissection at the Superior Mesenteric Artery: A Case Report

**DOI:** 10.3400/avd.cr.25-00011

**Published:** 2025-08-26

**Authors:** Kiyoshi Chiba, Yoshiki Yamasaki, Masahiro Tomita, Satoshi Kinebuchi, Takuma Fukunishi, Masahide Komagamine, Daijyun Tomimoto, Hiroshi Nishimaki, Kan Nawata

**Affiliations:** Department of Cardiovascular Surgery, St Marianna University School of Medicine, Kawasaki, Kanagawa, Japan

**Keywords:** hybrid surgery, multiple visceral aneurysms, isolated superior mesenteric artery dissection

## Abstract

A 59-year-old patient was undergoing careful monitoring of an isolated superior mesenteric artery dissection discovered 6 years prior. He was admitted after outpatient imaging revealed multiple visceral aneurysms including common hepatic and splenic artery aneurysms that had enlarged. Based on anatomical reasons and the past history, the splenic artery aneurysm was treated with endovascular therapy, while the common hepatic artery aneurysm was resected, and blood flow reconstruction was performed. The patient was discharged without any complications. Visceral artery aneurysms have diverse locations and morphologies, illustrating the importance of treatment strategies that consider the blood flow to the organs.

## Introduction

Visceral aneurysms are rare, but case reports exist in which they were diagnosed and treated through screening and testing for other diseases.^1,2)^ Endovascular treatment is considered the 1st choice for these aneurysms because of the favorable survival rate during the early stages of treatment.^3)^ However, in cases of multiple visceral aneurysms, it is important to devise a treatment strategy that considers the patient’s background and the blood flow to the organ.

We report a case in which hybrid treatment was performed for multiple visceral aneurysms in a patient with a history of dissection at the superior mesenteric artery (SMA).

## Case Report

**Figure 1:**
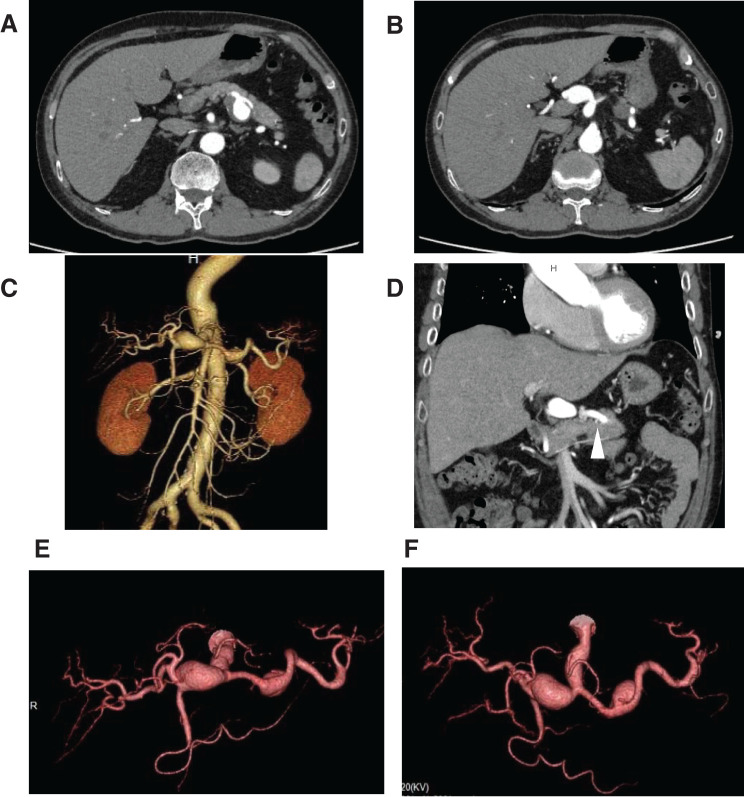
Fig. 1 CT and CT angiography. (**A**–**C**) CT angiography showed an SA aneurysm measuring 33 mm and a CHA aneurysm measuring 28 mm. The SMA was enlarged to 12 mm at the level of the jejunal branches, but no residual dissection was observed. (**D**) In the coronal view, the DPA branching near the aneurysm can be seen (arrowhead). (**E**) Left anterior oblique 9 and cranial 6 and (**F**) cranial 58. The CA had a normal diameter at the aortic bifurcation and at the branching points of the CHA and SA, but the CA was enlarged to 15 mm. CT: computed tomography; SA: splenic artery; CHA: common hepatic artery; SMA: superior mesenteric artery; CA: celiac artery; DPA: dorsal pancreatic artery

A 59-year-old man (height, 172 cm; weight, 58 kg; body mass index, 25.1 kg/m^2^) was transferred to our hospital 6 years prior with sudden abdominal pain and was hospitalized with a diagnosis of isolated SMA dissection. At that time, he was incidentally diagnosed with a celiac artery (CA) aneurysm measuring 15 mm, a common hepatic artery (CHA) aneurysm measuring 20 mm, and a splenic artery (SA) aneurysm measuring 28 mm, for which he was regularly observed at an outpatient clinic. During observation, the CHA aneurysm and the SA aneurysm expanded to 28 and 33 mm, respectively, and the patient was hospitalized for surgery. The patient had a history of hypertension and hyperlipidemia but no family history of vascular disease. The laboratory data on admission revealed no abnormalities. Chest-to- abdominal computed tomography (CT) angiography showed that the dissected SMA was slightly enlarged to 12 mm; however, the diameters of the pancreaticoduodenal artery and gastroduodenal artery (GDA) regions were normal, and the dorsal pancreatic artery (DPA) was visualized separately from the SA. The CA aneurysm developed 15 mm from the aortic bifurcation, and after temporarily returning to a normal diameter, the CHA aneurysm and SA aneurysm were observed (**Figs. 1A**–**1F**).

Based on preoperative imaging findings and clinical course, resection of the CHA with hepatic blood flow reconstruction was determined to be necessary. In summary, the CHA aneurysm extended to the confluence with the GDA, rendering it anatomically unsuitable for endovascular therapy. Furthermore, the middle colic artery bifurcation had already developed aneurysmal changes following an isolated SMA dissection. Therefore, we determined that hepatopetal blood flow must be preserved in preparation for future therapeutic interventions. For the SA aneurysm, which demonstrated sufficient anatomical landing zones both proximally and distally, the decision was made to treat it using a Viabahn stent graft (W. L. Gore & Associates, Flagstaff, AZ, USA).

The operation was performed in a hybrid operating room under general anesthesia. After laparotomy, the lesser omentum and gastroduodenal mesentery were opened, and the GDA, CHA, and proper hepatic artery (PHA) were exposed and taped. The left gastric artery was ligated and incised along with the gastric vein. Since the adhesions around the SA and CA were strong, exfoliation was minimized because of concerns about the development of pancreatic fistulas. Next, the abdominal aorta was exposed, and the great saphenous vein (GSV) was simultaneously harvested. The left brachial artery was exposed, and after a 4-Fr short sheath was placed, systemic heparin was administered.

**Figure 2:**
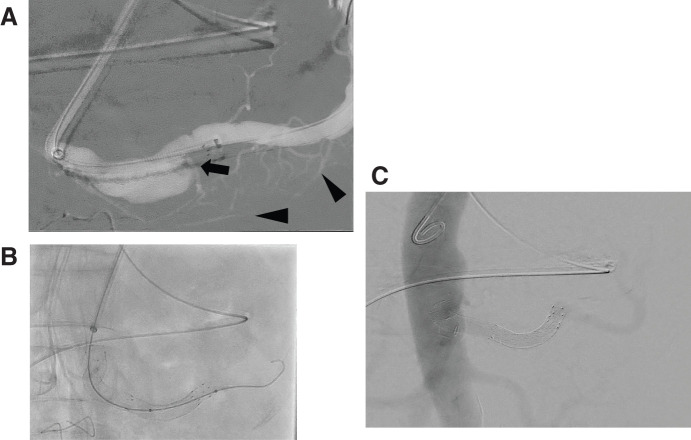
Fig. 2 Angiography of the SA aneurysm with graft. (**A**) The selective angiography of the SA revealed the great pancreatic artery and transverse pancreatic artery (arrowhead), in addition to the DPA (arrow). (**B** and **C**) A Viabahn stent graft (W. L. Gore & Associates, Flagstaff, AZ, USA) was visible from the distal side of the SA aneurysm to the branching point of the CA (7 mm × 5 cm + 9 mm × 10 cm). SA: splenic artery; CA: celiac artery; DPA: dorsal pancreatic artery

The SA was cannulated from the left brachial artery using a guidewire (Radifocus; Terumo Clinical Supply, Gifu, Japan), replaced with an SV wire (0.018-inch guidewire; Cordis, Tokyo, Japan), and an 8-Fr guiding catheter (STA; Medikit, Tokyo, Japan) was used to access the SA. The selective angiography of the SA revealed the great pancreatic artery and transverse pancreatic artery, in addition to the DPA identified on preoperative CT (**Fig. 2A**). The distance between the SA aneurysm and the bifurcation of the CA was measured using digital subtraction angiography (DSA), and a 7-mm × 5-cm Viabahn stent graft (W. L. Gore & Associates) was placed to preserve all vessels except for the DPA near the SA aneurysm. Additionally, a 9-mm × 10-cm Viabahn stent graft (W. L. Gore & Associates) was extended 5 mm on the aortic side to cover the CA aneurysm (**Figs. 2B** and **2C**).

It was confirmed that the CA and SA aneurysms had disappeared on DSA, and that the CHA aneurysm was contrasted via the SMA and pancreaticoduodenal artery (PDA). Next, the abdominal aorta was clamped below the renal artery, and an 8-mm end-to-side anastomosis was created with the GSV. The abdominal aorta was unclamped, and the twisting and length of the GSV were adjusted. A sufficient opening was created in the rough part of the mesentery to allow the GSV to pass through, and it was guided through the anterior aspect of the pancreas to the GDA. The proximal side of the CHA was double-ligated, and the PHA and GDA were clamped, and an 8-mm end-to-side anastomosis at the GDA with the GSV was created (warm ischemia time: 20 min). Subsequently, the PHA, GDA, and GSV were clamped to open the CHA aneurysm. The portal vein and aneurysm wall were exfoliated and excised, and vascular reconstruction was performed to ensure that the transition from the GDA to the PHA would not narrow. The final DSA confirmed that the blood flow from the anastomosed GSV to the PHA was satisfactory, and the Doppler flow meter showed no differences in blood flow at the same site. The abdomen was closed in the standard manner.

**Figure 3:**
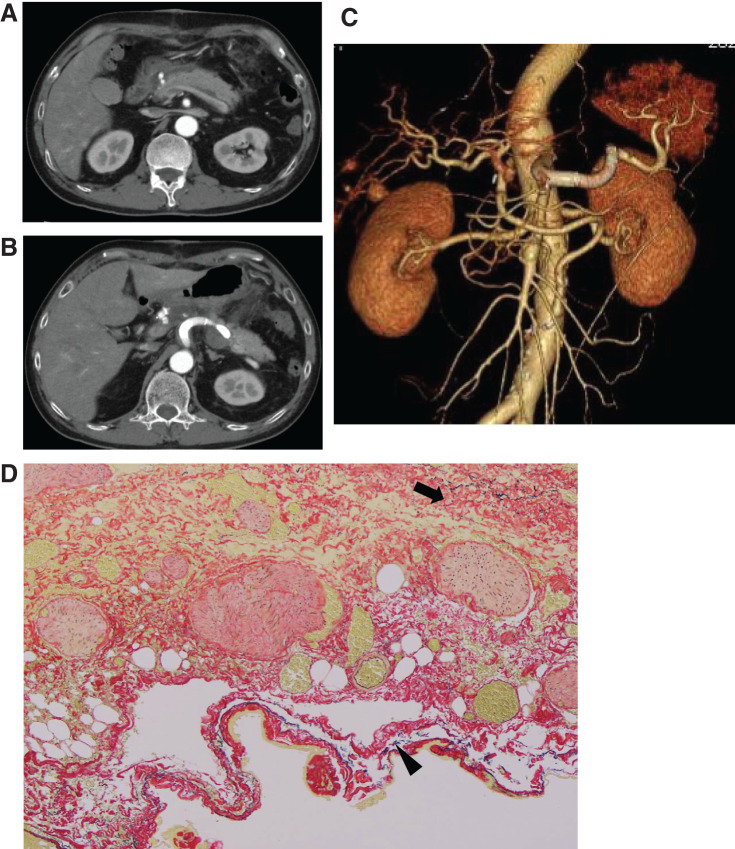
Fig. 3 Postoperative CT angiography and microscopic findings (Elastica van Gieson stain). (**A**–**C**) CT angiography performed 1 month postoperatively showed no abnormalities at the anastomosis site or the site of the aneurysm resection. Additionally, no endoleak was observed within the excluded CA aneurysm or SA aneurysm. (**D**) Microscopic findings showing the proliferation of collagen fibers (arrow) and the disruption of elastic fibers (arrowhead) were observed, suggesting that the formation of the aneurysm was attributable to atherosclerotic changes. CT: computed tomography; CA: celiac artery; SA: splenic artery

The postoperative course was uneventful, and the patient was discharged on postoperative day 8. A follow-up CT confirmed no abnormalities at the anastomosis site and no abnormalities in pancreatic contrast enhancement (**Figs. 3A**–**3C**).

## Discussion

Herein, we report a case of hybrid surgery for multiple visceral artery aneurysms. According to the Society for Vascular Surgery guidelines, asymptomatic SA aneurysms of 3 cm or larger and HA aneurysms of 2 cm or larger are indications for treatment, with endovascular intervention being the 1st choice if anatomically feasible.^3)^ Methods for endovascular treatment include isolation using covered stents, coil packing before and after the aneurysm, and the use of liquid embolic agents.^4,5)^

Typically, reconstruction of the SA is unnecessary. However, owing to the anticipated difficulties with exfoliation and the possibility of simultaneous treatment of the CA aneurysm, we chose endovascular treatment using a Viabahn stent graft (W. L. Gore & Associates) to achieve both blood flow preservation and exclusion of the aneurysm. Additionally, we selected a stent graft that could reliably seal the lumen, anticipating control of blood flow from the DPA branching from the non-aneurysmal SA.

Endovascular treatment using coil embolization was considered for the treatment of the CHA aneurysm. However, because of the proximity of the aneurysm’s distal end to the bifurcation of the PHA and GDA, a sufficient landing zone could not be secured. Therefore, direct surgical excision of the aneurysm was selected as the 1st option. We believe that placing the Viabahn stent graft (W. L. Gore & Associates) at the branching point of the CA in the treatment of the SA aneurysm contributes to control of blood flow on the proximal side of the CHA aneurysm, leading to more reliable management and minimizing the need for exfoliation.

However, opinions differ regarding the necessity of a bypass to maintain hepatic blood flow. In cases where coil embolization of the CA is performed during thoracic stent graft treatment for thoracoabdominal aortic aneurysms without vascular reconstruction, the incidence of visceral ischemia has been reported to be 9%.^6)^ On the other hand, risk factors for isolated SMA dissection include male gender, hypertension, smoking history, and Asian ethnicity.^7)^

And, cases have been reported where stenosis of the CA increases relative hepatofugal blood flow through the SMA, resulting in isolated SMA dissection.^8)^ In this case, the patient is a male with hypertension as a risk factor, who experienced isolated SMA dissection 6 years ago, and the branching points of the middle colic artery and jejunal branches were beginning to develop aneurysms. Although there are no direct reports, if only the excision of the CHA aneurysm had been performed, the increased blood flow through the SMA → PDA → GDA could have led to an increased shear stress on the SMA. In other words, if SMA dissection were to recur, there was a concern about the potential for extensive ischemia of the abdominal organs. Moreover, we chose to perform a bypass because we were concerned that treatment might become difficult if an existing aneurysm of the SMA enlarged or if a new aneurysm occurred in the pancreaticoduodenal arcade.^9)^

According to a report by Khan et al. involving 84 cases, the incidence of concomitant visceral aneurysms is 22%, with the CHA being the most frequent at 35%, followed by the CA at 25%. Additionally, the causes of the aneurysms are reported as atherosclerosis in 74% of cases, mycotic in 20%, and genetic or autoimmune diseases in 6%.^10^)^^ In this patient, although the imaging findings were atypical, we suspected segmental arterial mediolysis and conducted a pathological examination of the aneurysm wall. Microscopically, the proliferation of collagen fibers and the disruption of elastic fibers were observed, suggesting that the formation of the aneurysm was attributable to atherosclerotic changes, as shown with Elastica van Gieson stain (**Fig. 3D**).

In any case, maintaining hepatic blood flow and excising the aneurysm through direct surgery were prioritized based on the patient’s background.

## Conclusion

Hybrid treatments have been successfully used to treat multiple visceral arterial aneurysms. It is important to carefully consider the patient’s background and anatomical suitability when devising treatment strategies.
